# Isolation and Characterization of Heparan Sulfate from Human Lung Tissues †

**DOI:** 10.3390/molecules26185512

**Published:** 2021-09-10

**Authors:** Rupert Derler, Nikola Kitic, Tanja Gerlza, Andreas J. Kungl

**Affiliations:** 1 Institute of Pharmaceutical Sciences, University of Graz, Schubertstraße 1/1, 8010 Graz, Austria; rupert.derler@hotmail.com (R.D.); nikola.kitic@uni-graz.at (N.K.); tanja.gerlza@uni-graz.at (T.G.); 2 Antagonis Biotherapeutics GmbH, Strasserhofweg 77a, 8045 Graz, Austria

**Keywords:** glycosaminoglycans, heparan sulfate, lung, disaccharide composition, chemokines, chemokine/GAG interactions

## Abstract

Glycosaminoglycans are a class of linear, highly negatively charged, *O*-linked polysaccharides that are involved in many (patho)physiological processes. In vitro experimental investigations of such processes typically involve porcine-derived heparan sulfate (HS). Structural information about human, particularly organ-specific heparan sulfate, and how it compares with HS from other organisms, is very limited. In this study, heparan sulfate was isolated from human lung tissues derived from five donors and was characterized for their overall size distribution and disaccharide composition. The expression profiles of proteoglycans and HS-modifying enzymes was quantified in order to identify the major core proteins for HS. In addition, the binding affinities of human HS to two chemokines—CXCL8 and CCL2—were investigated, which represent important inflammatory mediators in lung pathologies. Our data revealed that syndecans are the predominant proteoglycan class in human lungs and that the disaccharide composition varies among individuals according to sex, age, and health stage (one of the donor lungs was accidentally discovered to contain a solid tumor). The compositional difference of the five human lung HS preparations affected chemokine binding affinities to various degrees, indicating selective immune cell responses depending on the relative chemokine–glycan affinities. This represents important new insights that could be translated into novel therapeutic concepts for individually treating lung immunological disorders via HS targets.

## 1. Introduction

Heparan sulfate is a member of the glycosaminoglycan (GAG) family of highly negatively charged unbranched polysaccharides. Other members of this group are heparin (HP), chondroitin sulfate (CS), dermatan sulfate (DS), keratan sulfate (KS), and hyaluronic acid (HA). HS consists of repeating disaccharide units of a hexose (D-glucuronic acid or its epimer L-iduronic acid) and an amino sugar (N-acetyl-D-glucosamine), which are 1→4 linked and usually reach a length of 50 to 200 disaccharide blocks. HS is either covalently attached to core proteins—forming proteoglycans (PGs), which can occur on the cell surface and in the extracellular matrix—or unattached as soluble molecules. PGs can be either transmembrane- or glycosylphosphatidylinositol-anchored, with attached Hs and/or CS chains [1]. After assembly by a complex synthetic machinery, the HS backbone is modified by many different enzymes in vivo. These modifications encompass sulfation at the 2-O-position of iduronic acid, sulfation at the 3-O- and 6-O-position of N-acetyl-glucosamine, and deacetylation/sulfation of the amino residue of N-acetyl-glucosamine. HS chain modification by acetylation and/or sulfation occurs in clusters that form so-called NA(high in acetylation)-, NS(high in N-sulfation)-, or NA/NS(intermediate)-domains [2,3]. Other modifications include epimerization of the glucuronic acid, desulfation of the 6-O-residue, or depolymerization of the HS chain (as reviewed in [3,4]). Therefore, HS chain modifications influence the protein-binding characteristics and subsequent physiological events triggered by binding [5,6,7,8,9,10,11]. The GAG bind to and present proteins at endothelial surfaces [12,13,14,15,16,17], which protects them against enzymatic degradation [18,19,20] and influences protein oligomerization [10,21,22,23]. Several hundred proteins are estimated to bind to HS [24,25]. The contribution of GAGs and chemokines to physiological and pathophysiological processes are manifold, as they are involved in cell differentiation and development [26,27,28], inflammation [29,30,31], cancer [32,33,34,35], neurodegenerative diseases [10,36,37], and host infection [36,37,38,39]. Additionally, HS serves as an essential structural molecule contributing to organ development, structural integrity, and endothelial and alveolar barrier function in the lung [40].

In the past, biochemical and physicochemical characterization of HS affinity and selectivity to different proteins frequently relied on the use of HP as a surrogate molecule for HS, which only poorly reflects HS’ variable degree of sulfation and epimerization [41,42]. An increasing number of studies tackled this issue by utilizing HS obtained from the bovine or porcine intestinal mucosa. However, structural information about HS in the lung is sparse, and it is unknown whether HS from bovine or porcine mucosa correctly reflects the GAG biology of the human lung. Findings in the literature describe the structural composition of murine [43,44,45] and rat [46,47] HS and reveal structural differences between species, individuals, and organs. Antibody approaches were conducted to analyze the HS composition of human lungs using immunohistochemistry. These studies revealed at least seven different HS epitopes in human lung sections [48]. To our knowledge, there is no study to date describing the isolation and further experimental use of human lung HS. As mentioned above, HS modification and overall structure has significant impact on its protein binding behavior and subsequent physiological events. Given the lack of knowledge about human HS, there is an obvious need for characterization of human HS in the lung, which we tackled in our study.

In this study, we isolated HS postmortem from human lungs of five donors using a previously published protocol [49] and elucidated their structure—overall chain length and disaccharide composition—as well as protein-binding behavior. For this purpose, we applied size exclusion chromatography (SEC-HPLC), enzymatic depolymerization, strong anion exchange chromatography (SAX-HPLC), and isothermal fluorescence titration (IFT). For the protein interaction studies of HS, we chose the chemokines CCL2 and CXCL8 [50,51] because of their implications in lung pathologies and their ability to attract different subsets of leukocytes. CCL2 (monocyte chemoattractant protein 1; MCP-1), a CC family chemokine, is an attractor and activator of monocytes and lymphocytes at sites of inflammation. It is an HS binding molecule [52] that exerts its function via activation of the CCR2 and CCR4 receptors [53,54]. CCL2 is involved in different disorders of the lung, such as inflammatory lung diseases [55,56,57], allergic asthma [58,59], pulmonary fibrosis [60,61], and lung cancer [62,63,64]. The chemokine CXCL8 (interleukin-8; IL-8) is a potent attractor of neutrophils, which is present in and contributes to inflammatory lung diseases [65,66,67]. It binds to HS [68] and signals upon activation of the CXCR1 and CXCR2 receptors [69,70].

## 2. Results

### 2.1. RT-qPCR

**Figure 1:**
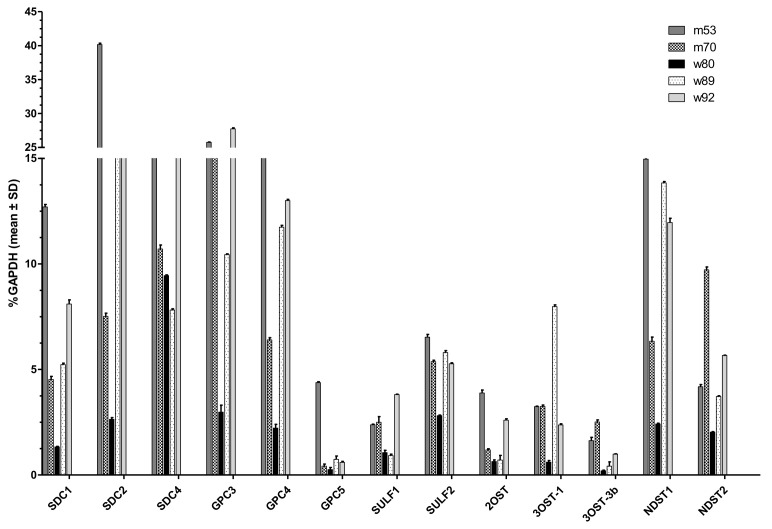
Expression profile of various genes in human lung tissues determined by RT-qPCR. Relative patient-specific mRNA expression levels of relevant syndecans (SDC) 1,2,4; glypicans (GPC) 3,4,5; sulfatases (SULF) 1,2; O-sulfotransferases (OST) 2OST, 3OST-1, 3 OST-3b; and N-sulfotransferase (NDST) 1,2. mRNA of five different donors are depicted in relation to the housekeeping gene glycerinaldehyd-3-phosphat-dehydrogenase (GAPDH) as mean ± SD with *n* = 3.

Due to the organ preparation from the deceased donors over several hours at RT, we checked RNA integrity via agarose gel electrophoresis before qPCR (data not shown). Figure 1 depicts those HS-relevant genes that could be detected in sufficient amounts compared with GAPDH. The most abundantly expressed proteoglycans were SDC1,2, 4, and GPC3 and 4, which reached up to 40% GAPDH expression. SDC3 and GPC1, 2, and 6 showed no expression above 2.5% GAPDH in any of the lung tissues. Furthermore, mRNA levels of heparan sulfate proteoglycan-modifying enzymes were analyzed. The dual activity enzyme N-deacetylase/N-sulfotransferase (NDST), which is responsible for removing acetyl groups from subsets of N-acetylglucosamine units and further transferring sulfuryl groups to these positions, depicted increased mRNA levels for NDST1 and 2, but not NDST3 and 4 (see Table 1). Sulfatases can modify the completed HS chain by 6-O-desulfation 1. SULF1 and 2 were both expressed up to around 6% GAPDH in the tested tissues. O-sulfotransferases (OST) were partly not expressed at all or showed very low expression. Of all tested OSTs, 2OST, 3OST-1, and 3OST-3B1 were most abundantly expressed. In total, 13 of the 28 tested genes showed expression levels of at least 2.5% GAPDH in at least one of the lung tissues. Comparison of gene expression profiles between different lungs revealed highly heterogenic expression patterns. To sum up, we could identify the main proteoglycans (SDC1,2,4, GPC3,4,5) and HS-modifying enzymes (SULF-1,2, 2OST, 3OST1, 3OST3B, NDST-1, and 2) expressed in lung tissues. Despite quantitative differences in the amount of expression, there was consistency in which genes were in principle actively expressed.

### 2.2. Chromatographic Characterization of HS Isolates

**Figure 2:**
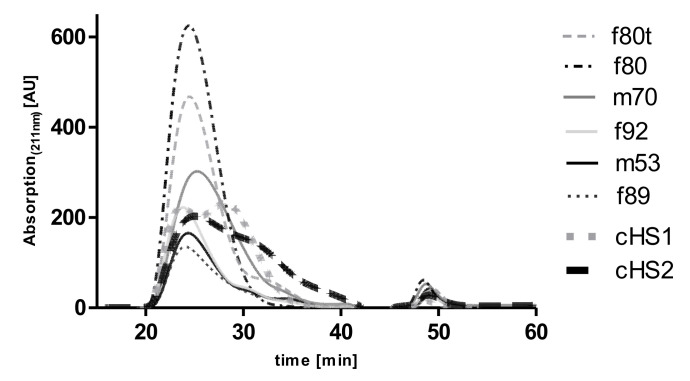
Size exclusion chromatogram of the different HS isolates from donor lungs (f80t, f80, m70, f92, m53, f89). Samples starting with f are from female patients, and those with m are from male, with the number depicting the age of the lung and the t identifying tumor lobe. Chromatograms are compared with two commercially available porcine-derived HS (cHS1 and cHS2). Data were recorded using a diode array detector, but the absorption maximum at 211 nm was used for the HS preparations and standards because of the highest sensitivity at this wavelength. The AUC of the HS preparations of the m53 and f89 differ significantly from the preparations of the other samples because different amounts of process-related impurities are expected to contribute to the absorption of the samples at 211 nm.

HS isolations from all five lungs displayed similar main retention times and peak widths in SEC, indicating similar chain lengths for all preparations (see Figure 2). The peak maxima were observed at around 24 to 26 min, followed by a small peak or shoulder at roughly 30 min, which indicated smaller chain length subfractions. As a comparison, two commercially available HS (cHS1 and cHS2) showed a bimodal distribution of chain lengths with one peak maximum eluting at 22 to 24 min and a second peak eluting at around 28 to 32 min. Taken together, our HS isolations represented the larger of two fractions, which could be observed in commercially available HS. Smaller chains seemed to be present in only relatively small amounts compared with those obtained from porcine origin.

**Figure 3:**
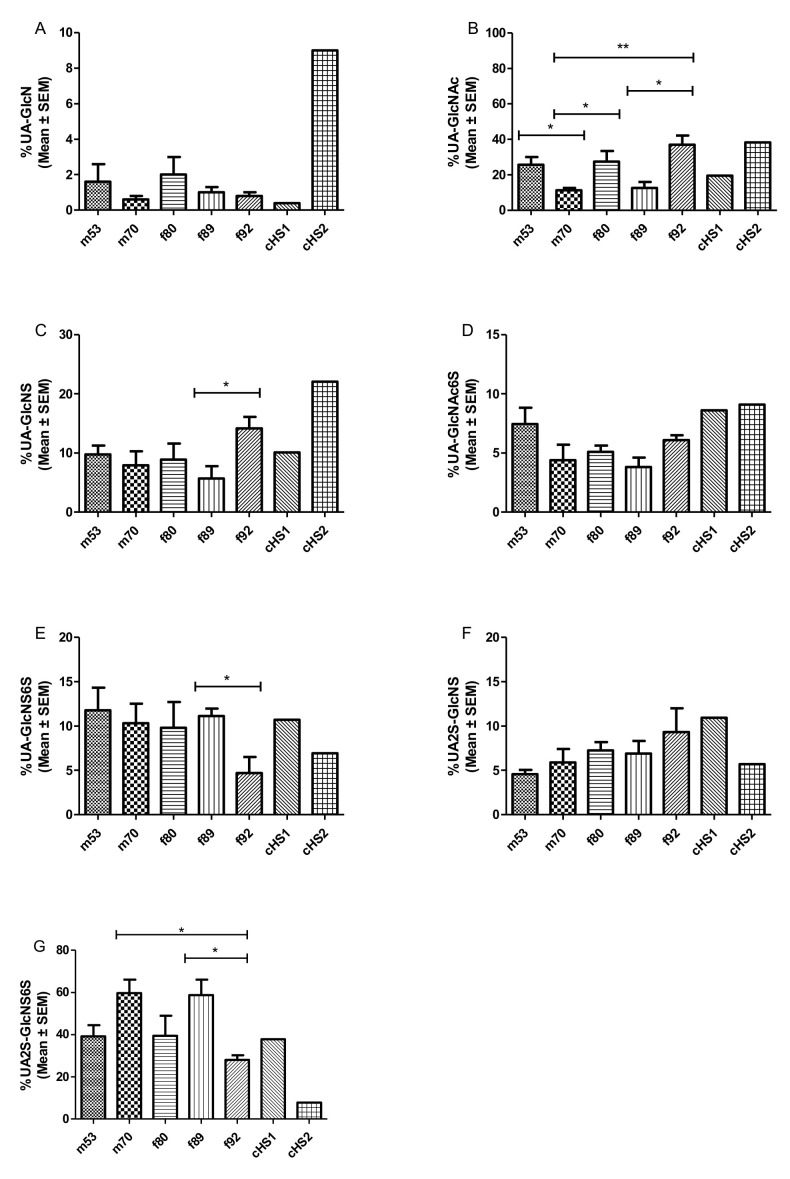
(**A**–**G**) Relative molar abundance (in percentage) of disaccharide composition of the different HS preparation from donor’s lungs, derived by SAX-HPLC, depicted as mean ± SEM. Data sets were compared using Student’s *t*-test. * *p* < 0.05, ** *p* < 0.01 was considered as statistically significant. (f refers to females, m to males, t to tumor lobe, and the number represents the donor’s age). Mean (± SEM) was calculated from three independent SAX-HPLC runs. The obtained AUCs were measured for each peak and compared with AUCs of commercially available HS disaccharide standards (Iduron).

Following enzymatic depolymerization and SAX HPLC, the resulting di-saccharides were separated by the degree and position of sulfate and acetyl modifications (according to reference standards). The degree of sulfation varied between the different lung preparations donors but also differed among individual preparations (see Figure 3 and Table 2). Table 2 shows the disaccharide range distributions of the individual GAG preparations and SAX HPLC runs, with data from a mouse disaccharide composition analysis. In Figure 3A–G, the different disaccharide compositions are shown as mean ± SEM for each donor’s lung. On the one hand, HS isolates of the m70 and f89 donor lungs showed an overall higher degree of sulfation compared with m53, f80, and f92, and they contained rather large amounts of the tri-sulfated disaccharide UA2S-GlcNS6S (59.7% and 58.8%). Moreover, m70 and f89 exhibited an overall lower degree of N-acetylated species (UA-GlcNAc 11.2% and 12.7%) and a lower number of free N-residues (0.6% and 1%). On the other hand, HS preparations of lungs m53, f80, and f92 were distinctly less sulfated. These isolates contained between 28.0% and 39.5% tri-sulfated disaccharides. Compared with the higher sulfated HS isolates, they displayed a higher degree of acetylated HS carbohydrates (0.7% up to 38.0%).

Working out statistically significant differences is difficult regarding the limited amount of HS that can be isolated from one lung sample. On top of the obvious trends in disaccharide composition displayed in Figure 3, significant differences were observed for the oldest patient f92, which showed a generally lower sulfation and higher acetylation content than f89. As these donors were approximately the same age and gender, these differences could be attributed to an inter-individual difference in GAG composition.

In comparison, commercially available HS preparations from porcine mucosa underline the possible heterogeneity in HS disaccharide composition (Table 2). cHS1 consists of relatively highly sulfated disaccharides, while cHS2 represents an HS with a relatively low grade of sulfation. The overall high sulfation of human HS isolates may seem surprising compared with HS disaccharides patterns obtained from murine lung tissues. In the literature, murine lung HS composition varies strongly [43,44,45] but generally shows a much lower degree of sulfation than our human isolates (see Table 2). According to these studies, the unsulfated, acetylated disaccharide UA-GlcNAc makes up between 45.3% and 63.5% of murine lung HS, whereas we found only 8.8% to 27.7% in human HS. Trisulfated HS disaccharide seems to only make up 2.7% to 9.7% of HS in mice, whereas we found 33.1% to 65.8% in human samples. It remains to be clarified where these discrepancies arise from. It is possible that sample cleanup or different workflows could enrich specific HS patterns [71]. There could, however, be a generally underestimated inter-species HS variability between humans and mice that has not been recognized yet.

### 2.3. IFT Measurements

**Figure 4:**
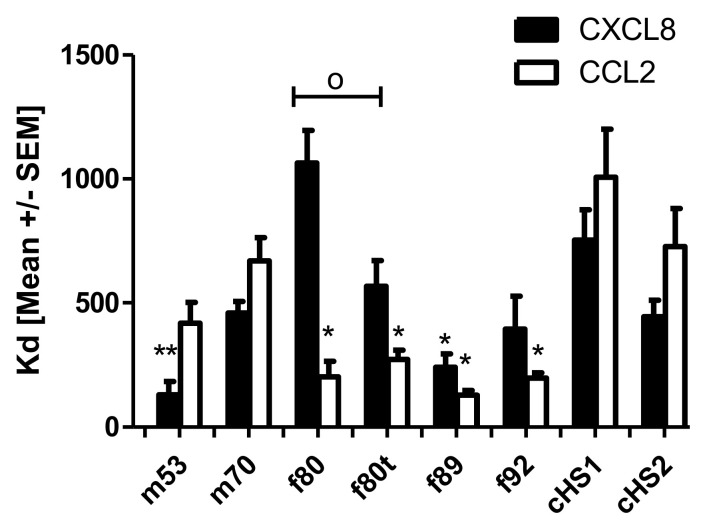
Isothermal fluorescence titration of CXCL8 and CCL2 with commercially available HS derived from porcine compared with HS prepared from lung tissue of different humans. With f for females, m for males, t for tumor lobe, and the number for the patient’s age. Data are shown as mean ± SEM of three measurements; groups were compared with the corresponding titration using Student´s *t*-test with * cHS1 or ^o^ between normal versus tumor lung lobe; */^o^ *p* < 0.05, ** *p* < 0.01.

Finally, we investigated the binding affinities of CCL2 and CXCL8 to different preparations of human lungs and commercially available GAGs using isothermal fluorescence titration (see Figure 4). Different binding patterns were observed for CCL2 and CXCL8. Highest binding affinities for CXCL8 were observed for m53 (K_d_ = 131 ± 53 nM), and K_d_ values decreased in order for f89 (242 ± 53 nM), f92 (396 ± 131 nM), cHS2 (446 ± 64 nM), m70 (460 ± 46 nM), cH1 (753 ± 122 nM), and f80 (1064 ± 202 nM). Interestingly the HS prepared from the tumor lobe showed a significantly increased binding affinity to CXCL8 (567 ± 124 nM) compared with the healthy lobe, which is comparable in binding to cHS2. For CCL2, the highest binding affinity was observed for f89 (K_d_ = 129 +/− 19) and decreased for f92 (198 ± 21 nM), f80 (203 ± 63 nM), m53 (419 ± 83 nM), m70 (669 ± 94 nM), cHS2 (727 ± 154 nM), and cHS1 (1006 ± 193 nM). In the case of CCL2, no difference is observed between the healthy and tumor lobe, indicating that there could be a selective change in GAG structure upon tumorigenesis that favors the binding of different chemokine species. Overall, with a few exceptions, human-derived HS showed higher binding affinities to human CXCL8 and CCL2 compared with porcine-derived HS.

## 3. Discussion

In the absence of direct sequencing methods for GAGs, structural—and thus functional—information about tissue-derived GAGs can only be inferred from disaccharide compositional and size distribution analyses. Here we found that the disaccharide composition of human lung-derived HS differed strongly among the individual donors and was, moreover, different from porcine (commercial) and murine lung-derived HS. We observed a higher binding affinity of human HS to human CXCL8 and CCL2 than porcine HS, which is in accordance with literature that already describes these differences for heparin [72,73]. These results lead us to hypothesize that the variance in disaccharide composition between individuals and species could be responsible for favorable, selective binding of specific human chemokines. In this context, it was shown previously for heparin that the composition between species and individuals differs significantly, leading to different binding patterns [74].

Considering individual GAG structures, it was noticed that both male samples did not show any UA-GlcN peaks, whereas these were present in all female samples. Since both male samples were younger, these differences could also be attributed to the different age and not only to gender. In addition, male samples displayed higher percentages of UA2S-GlcNS6S disaccharides, except for the f89 sample, which exhibited a vast amount of high sulfated disaccharides. From our relatively limited sample size (*n* = 5), we feel that the conclusions drawn are very valuable as a good first investigation, especially since the samples were able to cover an interesting range of ages and a balance of genders. We are currently trying to develop an MS-based method to characterize HS samples from very small tissue samples (such as from lung biopsies), thereby avoiding the preparation of HS from complete and intact human lungs. With smaller sample requirements, we hope to be able to improve the statistics of our studies in the future to *n* > 10.

We compared binding affinities of two commercially available HS samples of porcine origin with CCL2 and CXCL8, and we can conclude that cHS2 shows a higher binding affinity for both chemokines. In size-exclusion chromatography, we observed different elution peaks of these porcine HS compared with all human-derived samples, which indicates that chain lengths are different between species (Note: since the detailed preparation protocols of cHS1 and cHS2 are not available, the differences in SEC elution profiles may be partly due to modified preparation protocols of the commercial samples). HS from porcine mucosa eluted with two peaks, and only the main peak was also visible for our human preparations. When looking at the disaccharide composition, it is evident that cHS2 has a higher percentage of UAglcNAC, UA-GlcNS, and UA-GlcN, which seems to favor high binding affinity for both chemokines. In comparison, cHS1 has high amounts of highly sulfated disaccharides. By comparing the composition of these two HS samples with murine HS, we observed that cHS2 has more similarities to the mouse than cHS1: both contain high contents of UAglcNAC, UA-GlcNS, and UA-GlcN and only a low percentage of UA2S-GlcNS6S.

Additional RT-qPCR measurements showed highly heterogeneous expression of proteoglycans and HS-modifying enzymes. SDC-1, -2, and -4, as well as GPC-3 and -4 were the main expressed proteoglycans in the human tissues. SULF-1, SULF-2, 2OST, 3OST-1 3-OSTB1, NDST-1, and NDST-2 were the HS-modifying enzymes with the highest expression level. Biologically, polymerization of HS chains is accomplished by several enzymes of the exostosin family (EXT1 and EXT2) by alternate additions of GlcAβ1–4 and GlcNAcα1 to a maximal chain length of 200 disaccharides for HS. These growing chains are modified by a highly active dual-activity enzyme, the N-deacetylase/N-sulfotransferase (NDST), and other enzymes such as C5 epimerases O-sulfotransferases and by C5 epimerase, which add more flexibility to the nascent polysaccharide chain. These modifications form HS’s characteristic motifs, consisting of non-sulfated regions, mixed regions with alternating N-acetylated and N-sulfated sequences, and high sulfated regions [75]. These motifs are assumed to contribute to the GAG-side specificity of the protein–HS interaction [76,77]. The NS domain is reported to be between two and nine disaccharides in length, with even distribution along the chain and with N-sulfated content of HS to be around 30–40% [77,78]. The NS regions are assumed to adopt a rigid helical heparin-like symmetry in contrast to NA domains, which are more flexible.

The relative differences of K_d_ values of the two chemokines binding to the various HS preparations indicate preferential binding of each chemokine to a certain HS pattern. Since HS is assumed to be a co-receptor of chemokines, this finding further implies a remodeling of GAG patterns in lung tissues according to the chemokine-specific immune cells needed in case of certain immunological requirements. Particularly the differentiated GAG patterns observed in the tumor-containing lung lobe could point out different immune cell trafficking potentially unique to this disorder. If and how these findings, taken together with the corresponding disaccharide composition, could lead to novel therapeutic approaches to treat inflammatory and immunological disorders of the lung via GAGs remains to be investigated in the future.

## 4. Materials and Methods

### 4.1. Human Donor Lungs

Human donor lungs were received postmortem from the Medical University of Graz, Institute of Anatomy. The donors were two males, 52 and 70 years old (named m53 and m70), and three females, 80, 89, and 92 years old (named f80, f89, and f92). The lungs were histologically healthy, except one left lung lobe of the f80 lung, which contained metastasizing tumors (named f80t). The study was conducted in compliance with national, European, and international policies. This is particularly regulated by the Austrian Law for organ transplantation: §6 (1).

### 4.2. Recombinant Protein Production

Recombinant CCL2, which contained an N-terminal Met residue due to recombinant expression in *E. coli*, and recombinant CXCL8 were expressed in-house in *E. coli* and purified according to [53]. Amino acid sequences are depicted in Table 3.

### 4.3. RNA Extraction

Unless otherwise stated, all reagents were purchased from Merck (Darmstadt, Germany). Whole tissue RNA was extracted according to the Standard Protocol for the Isolation of RNA from the Quantitative Genomics Core Laboratory of the UTHSC-Houston (Houston, TX, USA), as published in [79]. One piece of the lung (~1 cm^3^) was ground in liquid nitrogen with pre-cooled mortar and pestle. The ground tissue was transferred to 15 mL centrifugal tubes, and 1 mL Tri reagent was added per 100 mg of tissue. After thorough homogenization, the sample was centrifuged at 12,000× *g* for 10 min and 4 °C. The supernatant was transferred to a clean centrifugal tube. An amount of 0.2 mL of CHCl_3_ was added to 1 mL of supernatant and homogenized. After incubation at RT for 2 min, the sample was centrifuged at 12,000× *g* for 15 min and 4 °C. The aqueous phase was transferred to a clean centrifugal tube, and 500 µL of isopropanol was added to every 600 µL of the aqueous phase. The sample was shaken for 10 sec, incubated for 10 min at RT, and subsequently centrifuged at 12,000× *g* for 10 min at RT. The supernatant was discarded, and the RNA pellet was washed with 500 µL of 75% EtOH. After centrifugation at 7500× *g* for 5 min at RT, the pellet was dried at RT. According to the manufacturer’s protocol, the pellet was subjected to further cleanup with the GenElute Mammalian Total RNA Miniprep Kit. Quality control of the isolation procedure and RNA integrity was performed by UV spectroscopy and agarose gel electrophoresis. For UV spectroscopy, the sample’s absorption was checked at emission wavelengths of 260 and 280 nm. QC of RNA integrity was performed by 1% agarose gel in TAE buffer containing SYBR Safe stain (Thermo Fisher, Waltham, MA, USA). Next, 5 µL of extracted RNA was prepared with 1 µL of 6X orange loading dye (Thermo Fisher, Waltham, MA, USA). An amount of 1 µL of 1 kbp O’Gene ruler (Thermo Fisher, Waltham, MA, USA) was applied as DNA size standard. The gel was run for 50 min at 80 V constant current. Visualization was carried out in a Chemidoc+ imaging system (Bio-Rad, Berkeley, CA, USA) with an excitation wavelength of 280 nm and emission wavelength of 530 nm.

### 4.4. Relative RT-qPCR

All reagents and primers used for RT-qPCR were purchased from Thermo Fisher (Waltham, MA, USA). An amount of 2 µg of RNA of each sample was reverse transcribed using the High Capacity cDNA Reverse Transcription Kit, according to the manufacturer’s protocol without the RNAse inhibitors. The Power SYBR Green PCR MasterMix was used for the qPCR reaction, according to the manufacturer’s protocol. See Table 4 for an overview of the investigated genes, the primer sequences and the mRNA accession number used to design primers.A no-template control and a no reverse transcription control were added to each reaction plate. The qPCR reaction and signal detection was performed on an Applied Biosystems 7300 Real-Time PCR System (Waltham, MO, USA). The Thermo cycle program consisted of an initial denaturation step (10 min at 95 °C), followed by 40 cycles of denaturation (15 s at 95 °C), primer annealing (30 s at 60 °C), and elongation (1 min at 72 °C). In the end, one final dissociation step (system default) was added. In the end a melting curve was performed to show specificity for the primers used in this investigation. For all primers, one sharp peak was obtained, proving that the primers only amplified one main product (data not shown). The 7000 system SDS software (Applied Biosystems, V 1.2.3) was used for data visualization and analysis A manual Ct threshold of 0.3 and the auto-baseline correction were applied. Each sample was measured thrice, and gene expression was calculated using 2^−0-ΔCt^ calculation, and donors were compared relative to each other to see the difference between individuals. Values were normalized to GAPDH housekeeping expression and are shown as mean ± SD.

### 4.5. Isolation of Heparan Sulfate

HS was isolated using a previously published protocol [49]. The lung tissues were stored at −80 °C. For HS isolation, the tissue was thawed overnight at 4 °C and cut to pieces of approximately 1 cm^3^. For each g of tissue, 4 mL of a 50 mM phosphate buffer (pH 6.5, 2 mM EDTA, 2 mM L-cysteine) was added and homogenized with a blender. For each g of tissue, 15 U of papain (Merck Darmstadt, Germany)) was added to the homogenate and incubated at 65 °C for 72 h, shaking at 120 rpm. After cooling on ice, the sample was set to 0.2 M NaOH and 1% (*w*/*v*) NaBH_4_ (from Merck (Darmstadt, Germany)) and incubated at 4 °C overnight to reductively eliminate the O-linked glycans from the peptide backbone. The reaction mixture was neutralized with glacial acetic acid and subsequently extracted with an equal volume of CHCl_3_. The aqueous phase was taken and centrifuged at 17 k× *g* for 40 min at room temperature. The supernatant was diluted 1:1.2 with deionized water to decrease the ionic strength of the solution. The solution was loaded onto a 10 mL column packed with DEAE Sepharose (Cytiva, Marlborough, MA, USA) connected to an ÄKTA prime FPLC system (Cytiva, Marlborough, MA, USA) at a 2 mL/min flow rate. After loading, the column was washed with 100 mL of 0.15 M NaCl in 0.1 M Trizma-HCl buffer (pH 7.0), and the GAGs were eluted with 50 mL of 2.0 M NaCl in 0.1 M Trizma-HCl buffer (pH 7.0). Proteins and DNA were precipitated from the eluate adding 100% (*w*/*v*) TCA (Merck Darmstadt, Germany) to a final concentration of 10% (*v*/*v*) and incubated on ice for 30 min. After centrifugation at 17 k× *g* for 30 min at room temperature, the supernatant was taken, neutralized with NaOH, and rebuffered with 50 mM Trizma-HCl (pH 8.0, 60 mM sodium acetate, 0.02% BSA) using 50 mL AMICON 3 kDa cut-off centrifugal filter devices (Merck Millipore). CS and DS were removed from the isolations by enzymatic depolymerization with 50 mIU chondroitinase ABC (Merck Darmstadt, Germany) for 72 h at 37 °C while shaking at 150 rpm. BSA and chondroitinase were removed by TCA treatment, centrifugation, and neutralization, as described above. The supernatant was loaded onto a 1 mL DEAE Sepharose column, washed with 10 mL of 0.15 M NaCl Trizma-HCl buffer (as above), and the HS was eluted with 2.0 M NaCl in 0.1 M Trizma-HCl buffer (as above). The eluate was desalted and concentrated using 15 mL AMICON MW 3 kDa cut-off centrifugal filter devices (Merck Darmstadt, Germany)) with deionized water. The retentate was lyophilized, and the dried HS was weighed.

### 4.6. Size Exclusion Chromatography

The samples were separated at a flow rate of 0.5 mL/min for 50 min. An amount of 100 µg of each isolated HS sample, as well as of commercially available HS (cHS) from Iduron Ltd. (Cheshire, UK) and Celsus Laboratories Inc. (Cincinnati, OH, USA) was diluted in 50 µL of running buffer (1 M NaCl buffer containing 0.1 M Trizma-HCl, pH 7.0) and subjected to size exclusion chromatography (SEC) on a VWR Hitachi L-2130 Elite LaChrom HPLC System connected to an L-2450 Diode Array Detector, an L-2200 Autosampler (VWR, Radnor, PA, USA), and a Superdex 75 size exclusion column (Merck Darmstadt, Germany). The wavelength of 211 nm showed an absorption maximum for the HS preparations and standards and was chosen as the detection wavelength.

### 4.7. Enzymatic Depolymerization of HS

HS was enzymatically depolymerized by heparinases I, II, and III (Iduron, Cheshire, UK). An amount of 100 µg of each HS isolation and cHS1 and cHS2 was dissolved in 20 µL of 100 mM sodium acetate buffer (pH 7.5; 2 mM calcium acetate) containing 5 mIU each of heparinase I, II, and III. The mixture was incubated for 48 h at 37 °C and shaken at 120 rpm.

### 4.8. Strong Anion Exchange Chromatography

An amount of 50 µg of enzymatically depolymerized HS sample was separated by strong anion exchange chromatography on a Shimadzu UFLC- HPLC system connected to a UV-VIS detector (20AD, Kyoto, Japan), as described earlier [49]. All preparations except for the tumor lobe were prepared thrice, and SAX was measured once for each preparation using a ProPac PA1 analytical LC column (Thermo Fisher, Waltham, MA, USA). Because the tumor lobe material was limited, only a single preparation could be used for disaccharide composition analysis. cHS 1 and 2 from Iduron (Cheshire, UK) and Celsus (Cincinnati, OH, USA) was depolymerized once, and disaccharide composition was in the usual obtained range (data not shown). For relative quantification of HS disaccharide composition, the obtained AUCs were compared with AUCs of commercially available HS disaccharide standards (Iduron, Cheshire, UK). Therefore, AUC from the standards were divided by the applied mol and using these the AUC from the samples were back-calculated for their mol, and further relative abundancy was calculated by dividing through the mol sum of the different obtained disaccharide specimens. The mean of three different preparations was calculated by adding the scores together and then dividing by the number of scores.

### 4.9. Isothermal Fluorescence Titration

The binding affinity of HS to recombinant CCL2 and CXCL8, was measured by isothermal fluorescence titration, as already described [49,80]. Fluorescence quenching due to protein GAG interaction was measured under increasing GAG concentrations. The normalized mean changes in fluorescence were plotted against the GAG concentration and fitted by nonlinear regression. The K_d_ constants were derived from an equation for bimolecular association [81]. Equation (1): Calculation of K_d_ values.

(1) $$F=F_{i}+F_{\max}\left[\;\frac{K_{d}+\left[\mathrm{protein}\right]+\left[\mathrm{ligand}\right]-\sqrt{{\left(K_{d}+\left[\mathrm{protein}\right]+\left[\mathrm{ligand}\right]\right)}^{2}-4\left[\mathrm{protein}\right]\left[\mathrm{ligand}\right]}}{2\left[\mathrm{protein}\right]}\right]$$

### 4.10. Statistical Analysis

All data shown for IFT are reported as mean ± SEM (standard error of the mean) for *n* observations. Statistical analysis was performed using Graphpad Prism 5.0 using Student’s *t*-test comparing individual titrations with the matching cHS1 (CXCL8 and CCL2, respectively). * *p* < 0.05 and ** *p* < 0.01 were considered as statistically significant. All measurements were performed in triplicates.

## Figures and Tables

**Table 1:** Relative gene expression profile (% GAPDH) of different HS isolates from human lung in relation to GAPDH expression. Relative patient-specific mRNA expression levels of syndecans (SDC) 1,2,3,4; glypicans (GPC) 1,2 3,4,5; sulfatase (SULF) 1,2; O-sulfotransferases (OST) 2OST, 3OST-1,3OST-2, 3OST-3A1, 3OST3b, 3OST 4–6, 6OST 1–3; heparanase (HPSE); and N-sulfotransferase (NDST) 1–4. mRNA of five different donors are depicted in relation to the housekeeping gene GAPDH as mean ± SD with *n* = 3.

	m53		m70		f80		f89		f92	
	Mean	SD	Mean	SD	Mean	SD	Mean	SD	Mean	SD
SDC1	12.70	0.12	4.53	0.14	1.33	0.03	5.23	0.07	8.10	0.20
SDC2	40.15	0.20	7.52	0.15	2.64	0.08	19.48	0.04	19.17	0.19
SDC3	1.36	0.08	1.14	0.09	0.18	0.16	0.49	0.05	1.26	0.12
SDC4	16.23	0.06	10.71	0.19	9.45	0.03	7.82	0.06	23.93	0.07
GPC1	0.36	0.16	0.28	0.02	0.05	0.09	0.25	0.32	0.67	0.02
GPC2	0.05	0.30	0.07	0.09	0.01	0.54	0.01	0.46	0.02	0.16
GPC3	25.76	0.04	24.37	0.07	2.98	0.33	10.44	0.04	27.74	0.14
GPC4	16.57	0.09	6.40	0.11	2.22	0.18	11.74	0.09	13.00	0.06
GPC5	4.38	0.04	0.42	0.10	0.26	0.10	0.74	0.16	0.60	0.05
GPC6	0.85	0.22	0.91	0.24	0.24	0.19	1.02	0.14	2.20	0.16
SULF1	2.38	0.03	2.50	0.27	1.06	0.11	0.92	0.07	3.81	0.02
SULF2	6.53	0.13	5.37	0.07	2.81	0.03	5.80	0.09	5.27	0.05
2OST	3.89	0.14	1.19	0.05	0.64	0.07	0.71	0.22	2.60	0.06
3OST-1	3.24	0.03	3.25	0.07	0.61	0.07	7.98	0.08	2.37	0.06
3OST-2	0.23	0.03	0.79	0.05	0.08	0.11	0.42	0.37	1.09	0.03
3OST-3A1	0.13	0.15	0.11	0.22	0.01	0.28	0.00	0.67	0.06	0.13
3OST-3B1	1.63	0.15	2.51	0.10	0.20	0.03	0.42	0.20	0.99	0.01
3OST-4	0.34	0.18	0.18	0.16	0.04	0.04	0.05	0.18	0.06	0.37
3OST-5	0.07	0.45	0.02	1.74	0.01	0.24	0.03	0.35	0.03	0.24
OST6	0.17	0.28	0.03	0.23	0.02	0.11	0.02	0.96	0.06	0.03
6OST-1	0.64	0.11	1.30	0.54	0.01	0.56	0.36	0.54	1.41	0.06
6OST-2	0.64	0.12	0.22	0.06	0.23	0.16	0.24	0.13	0.27	0.22
6OST-3	0.04	0.27	0.02	0.27	0.19	0.04	0.01	0.80	0.05	0.33
HPSE	0.38	0.18	0.41	0.08	0.05	0.21	0.84	0.10	0.46	0.33
NDST1	14.97	0.14	6.34	0.19	2.42	0.03	13.84	0.07	11.96	0.20
NDST2	4.18	0.10	9.72	0.14	2.03	0.03	3.72	0.03	5.66	0.02
NDST3	0.02	0.32	0.00	1.15	0.00	0.26	0.00	1.10	1.64	0.12
NDST4	0.04	0.55	0.00	1.03	0.05	0.18	0.01	0.53	0.01	0.54

**Table 2:** Molar HS disaccharide percentage range found in five different human lung isolates compared with murine lung HS and commercially porcine-derived HS. All preparations were prepared thrice, and the disaccharide composition percentage range is listed. With f for females, m for males, and the number for the patient’s age, n.d. for not detected.

	m53	m70	f80	f89	f92	cHS1 ^1^	cHS2 ^1^	HS Mouse	HS Mouse ^2^
UA-GlcN	0.2–3.5	0.2–0.8	0.6–4.2	0.5–1.33	1.4–1.9	0.4	9.0	3.6	n.d.
UA-GlcNAC	17.2–31.1	8.9–13.4	25.8–39	8.8–19	27.7–44.3	19.6	38.2	4.4	45.3–63.5
UA-GlcNS	6.8–11.3	4.9–12.6	4.2–9.3	3.1–9.7	11.7–18.2	10.1	22.1	5.4	10.2–21.4
UA-GlcNAc6S	4.9–9.6	2.1–6.5	2.2–5.6	2.9–5.36	6.4–7.7	8.6	9.1	5.2	2.1–8.5
UA2S-GlcNAc	n.d.	n.d.	n.d.	n.d.	n.d.	1.8	1.1	1.2	n.d.–4.9
UA-GlcNS6S	7.5–16.3	5.8–12.9	4.8–14.84	9.7–12.7	3.5–9.1	10.7	6.9	15.7	3.5–6.5
UA2S-GlcNS	4–5.6	3.9–8.76	6.4–9.5	5.1–9.7	5.4–10.4	10.9	5.7	7.8	6.0–17.2
UA2S-GlcNAc6S	n.d.	n.d.	n.d.	n.d.	n.d.	n.d.	n.d.	n.d.	n.d.–0.3
UA2S-GlcNS6S	28.9–46.2	59.7–70.7	26.9–58.4	44–65.8	25.2–33.1	37.9	7.8	56.8	2.7–9.7

^1^ Commercially available HS from porcine mucosa; ^2^ Data from three different studies conducted in mice [43,44,45].

**Table 3:** Amino acid sequences of the recombinantly expressed chemokines used in this study.

Protein	Amino Acid Sequence
CCL2	MQPDAINAPVT CCYNFTNRKI SVQRLASYRR ITSSKCPKEA VIFKTIVAKE ICADPKQKWV QDSMDHLDKQ TQTPKT
CXCL8	SAKELRCQCI KTYSKPFHPK FIKELRVIES GPHCANTEII VKLSDGRELC LDPKENWVQR VVEKFLKRAE NS

**Table 4:** Genes investigated by RT-qPCR and their respective primer sequences. Gene accession number used for primer design and position of the amplicon is depicted.

	Protein	Primer Sequences	Amplicon
GAPDH	Glycerinaldehyde-3-phospate-dehydrogenase	5′ ATGTTCGTCATGGGTGTGAA 3′ GTCTTCTGGGTGGCAGTGAT	NM_001289746.2 704–876 = 173 bp
SDC-1	Syndecan-1	5′ GGAGCAGGACTTCACCTTTG 3′ TACAGCATGAAACCCACCAG	NM_002997.4 920–1126 = 207 bp
SDC-2	Syndecan-2	5′ GCTGCTCCAAAAGTGGAAAC 3′ CAGCAATGACAGCTGCTAGG	BC049836.1 580–790 = 211 bp
SDC-3	Syndecan-3	5′ GAGCCTGACATCCCTGAGAG 3′ CCCACAGCTACCACCTCATT	NM_014654.4 971–1182 = 212 bp
SDC-4	Syndecan-4	5′ GAGCCCTACCAGACGATGAG 3′ CAGTGCTGGACATTGACACC	BC030805.1 159–443 = 285 bp
GPC-1	Glypican-1	5′ AGCGAGATGGAGGAGAACCT 3′ CTGAGTACAGGTCCCGGAAG	BC051279.1 432–660 = 229 bp
GPC-2	Glypican-2	5′ ACTGGGACACGACCTGGAC 3′ CCCCAGAACCATCCCTTCTA	NM_152742.3 1637–1791 = 155 bp
GPC-3	Glypican-3	5′ GGCAAGTTATGTGCCCATTC 3′ ATGTAGCCAGGCAAAGCACT	KX533474.1 1389–1579 = 191 bp
GPC-4	Glypican-4	5′ ATGGTGGCAGAGAGGCTAGA 3′ GGAACGAGAAATTCGTCCAG	AF030186.1 1101–1277 = 177 bp
GPC-5	Glypican-5	5′ AAGCCCAGTCTGGAAATCCT 3′ TCACAGTCCCCACTGACTTG	AF001462.1 1394–1577 = 184 bp
GPC-6	Glypican-6	5′ CACGTTTCAGGCCCTACAAT 3′ GTTCCAGCATTCCTCCTCGT	AF105267.1 1670–1875 = 188 bp
NDST-1	Bifunctional heparan sulfate N-deacetylase/N-sulfotransferase 1	5′ TCACCTTCAACCTGGGCTAC 3′ ACGGACTGGTTGTGGAAAAG	NM_001543.5 1538–1694 = 157 bp
NDST-2	Bifunctional heparan sulfate N-deacetylase/N-sulfotransferase 2	5′ATCATCACAGTGCTCACCAACCCT 3′AGCCAGCGTTGTAGATGGGTAGAA	NM_003635.4 2663–2862 = 200 bp
NDST-3	Bifunctional heparan sulfate N-deacetylase/N-sulfotransferase 3	5′ TCAGGGAAGAGGCTGACATT 3′ ATCCACAGACCCCAACAGAC	NM_004784.3 1139–1381 = 243 bp
NDST-4	Bifunctional heparan sulfate N-deacetylase/N-sulfotransferase 4	5′ CCACCTCTTCCACAACGAGT 3′ GGCAGGTTTCAGATGTGGAT	NM_022569.3 1581–1794 = 214 bp
2OST	Heparan sulfate 2-O-sulfotransferase 1	5′ TGGAAAGAGATGAAACCAGGA 3′ CAGAGCTTCTCTGGAGCACA	NM_012262.4 793–1046 = 254 bp
3OST-1	Heparan sulfate glucosamine 3-O-sulfotransferase 1	5′ TCCAAAAGGTCGAGAGGTTCCT 3′ AGGCAGTAAAAGCCCTTGGTTT	NM_005114.4 987–1074 = 88 bp
3OST-2	Heparan sulfate glucosamine 3-O-sulfotransferase 2	5′ CCCCACTTCTTTGACAGGAA 3′ TGTCTCGGGACATGTTGAAG	NM_006043.2 888–1041 = 154 bp
3OST3-A1	Heparan sulfate glucosamine 3-O-sulfotransferase 3A1	5′ GACTTTGGCTGGGATGGATA 3′ GATCCACGTGTTTGGTGTTG	NM_006042.3 2001–2203 = 203 bp
3OST3-B1	Heparan sulfate glucosamine 3-O-sulfotransferase 3B1	5′ GCTGCCTAGCCACACTCTTT 3′ GGGAGACCCAAGACAAGACA	NM_006041.3 1737–1979 = 243 bp
3OST-4	Heparan sulfate glucosamine 3-O-sulfotransferase 4	5′ TACGAAAAGGGGTTGGAGTG 3′ TAGTCAGAGATGGCCCTGGT	NM_006040.3 1168–1352 = 185 bp
3OST-5	Heparan sulfate glucosamine 3-O-sulfotransferase 5	5′ AGTTGGGAGCTTGGATAGGC 3′ CCTTTCCTCACCCCAATGAT	NM_153612.4 1257–1469 = 213 bp
3OST-6	Heparan sulfate glucosamine 3-O-sulfotransferase 6	5′ CATCGTTGGCGTGAAGAAG 3′ ACGAAGTAGCTGGGGGTCTT	NM_001009606.4 374–568 = 195 bp
6OST-1	Heparan sulfate 6-O-sulfotransferase 1	5′ AAGAAGTGCACCTGCTACC 3′ CGCCCATCACACATATGCAA	NM_004807.3 672–946 = 275 bp
6OST-2	Heparan sulfate 6-O-sulfotransferase 2	5′ CCGTCCAGGTGGAGGATTT 3′ GACCAGTCATCGCCAGTGTA	NM_001077188.2 1331–1623 = 293 bp
6OST-3	Heparan sulfate 6-O-sulfotransferase 3	5′ CAAGAAGGAGACGTGGCTCT 3′ GGGCTTCTTCCATCACACAT	NM_153456.4 1326–1583 = 258 bp
SULF-1	Extracellular sulfatase Sulf-1	5′ ATACTCGGCAGACACGTTCC 3′ CTCTGGCCGATTGGTACAGT	NM_001128205.2 2285–2581 = 297 bp
SULF-2	Extracellular sulfatase Sulf-2	5′ ACACGTACTGGTGCATGAGG 3′ GCTTGTAACCCTTGCAGCTC	NM_018837.4 2626–2823 = 198 bp
HPSE	Heparanase	5′ CTGGCTTTATGTGGCTGGAT 3′ GCTTGCCATTAACACCTTGG	NM_006665.6 1219–1403 = 185 bp

## Sample Availability

Lung samples of the compounds are not available from the authors. Samples of the compounds CXCL8 or CCL2 are available from the authors.
